# A slender symbiotic goby hiding in burrows of mud shrimp *Austinogebia edulis* in western Taiwan

**DOI:** 10.1371/journal.pone.0219815

**Published:** 2019-07-22

**Authors:** Li-Chun Tseng, Shih-Pin Huang, Shagnika Das, I-Shiung Chen, Kwang-Tsao Shao, Jiang-Shiou Hwang

**Affiliations:** 1 Institute of Marine Biology, College of Life Sciences, National Taiwan Ocean University, Keelung, Taiwan; 2 Center of Excellence for the Oceans, National Taiwan Ocean University, Keelung, Taiwan; 3 Biodiversity Research Center, Academia Sinica, Taipei, Taiwan; 4 Univ. Lille, CNRS, Univ. Littoral Cote d’Opale, UMR 8187, LOG, Laboratoire d’Oceanologie et de Geosciences, Wimereux, France; Tanzania Fisheries Research Institute, UNITED REPUBLIC OF TANZANIA

## Abstract

The present study recorded the population of the goby fish (Perciformes: Gobiidae), *Eutaeniichthys* cf. *gilli* Jordan & Snyder, 1901, from the tunnel burrowed by the mud shrimp *Austinogebia edulis* Ngo-Ho and Chan, 1992 in a mudflat in Shengang and Wangong of Changhua County, western Taiwan. This finding is not only a new record of the genus in Taiwan, it is also the first record of this species in a mudflat near an industrial park. In total, 56 individuals of *E*. cf. *gilli* were collected from June 2016 to September 2018. Morphological traits of males and females were measured. The resin casting method trapped bodies of *E*. cf. *gilli* that were present in the tunnel burrow and proved that the fish inhabits burrows of the mud shrimp *A*. *edulis*. In addition, a species of snapping shrimp was also found in the same tunnel. Symbiotic interaction may occur between *E*. cf. *gilli*, *A*. *eduli* and the snapping shrimp. The China Coastal Current (CCC) runs along the coastlines of Japan, Korea, China, and reaches western Taiwan during the northeast monsoon period. The CCC, therefore, might play an important role in the biogeographic distribution of *E*. cf. *gilli* in the western Pacific Ocean. Since *E*. cf. *gilli* is listed in the Red List as an endangered species of Japan for many years, Taiwan waters may provide a refuge for this fish species warranting a broader investigation. Since Taiwan is some distance away from the previously recorded locations in Japan, Korea, the Yellow Sea, and the Bohai Sea, a phylogenic analysis is warranted for population and species differentiation in the future.

## Introduction

In order to develop the industry and extend land use towards the ocean, the use of limited land, large-scale land reclamation was carried out in wetlands along the western coast of Taiwan [1]. Such man-made reclamations change the coastal environment, causing habitat fragmentation, reducing the resources of prey, and causing contaminations through pollution [2, 3]. They will, therefore, impact the coastal ecosystems and endanger organisms that inhabit the area. Examples include endangered populations of the Chinese white dolphin, *Sousa chinensis* (Osbeck, 1765) [4], changes in the breeding habitat of little tern, *Sterna albifrons* [5], accumulation of heavy metals in soldier crab, *Mictyris brevidactylus* [6], and decrease in the density of mud shrimp, *Austinogebia edulis* (Ngoc-Ho & Chan, 1992; synonymy: *Upogebia edulis* Ngoc-Ho & Chan, 1992) [7].

**Fig 1:**
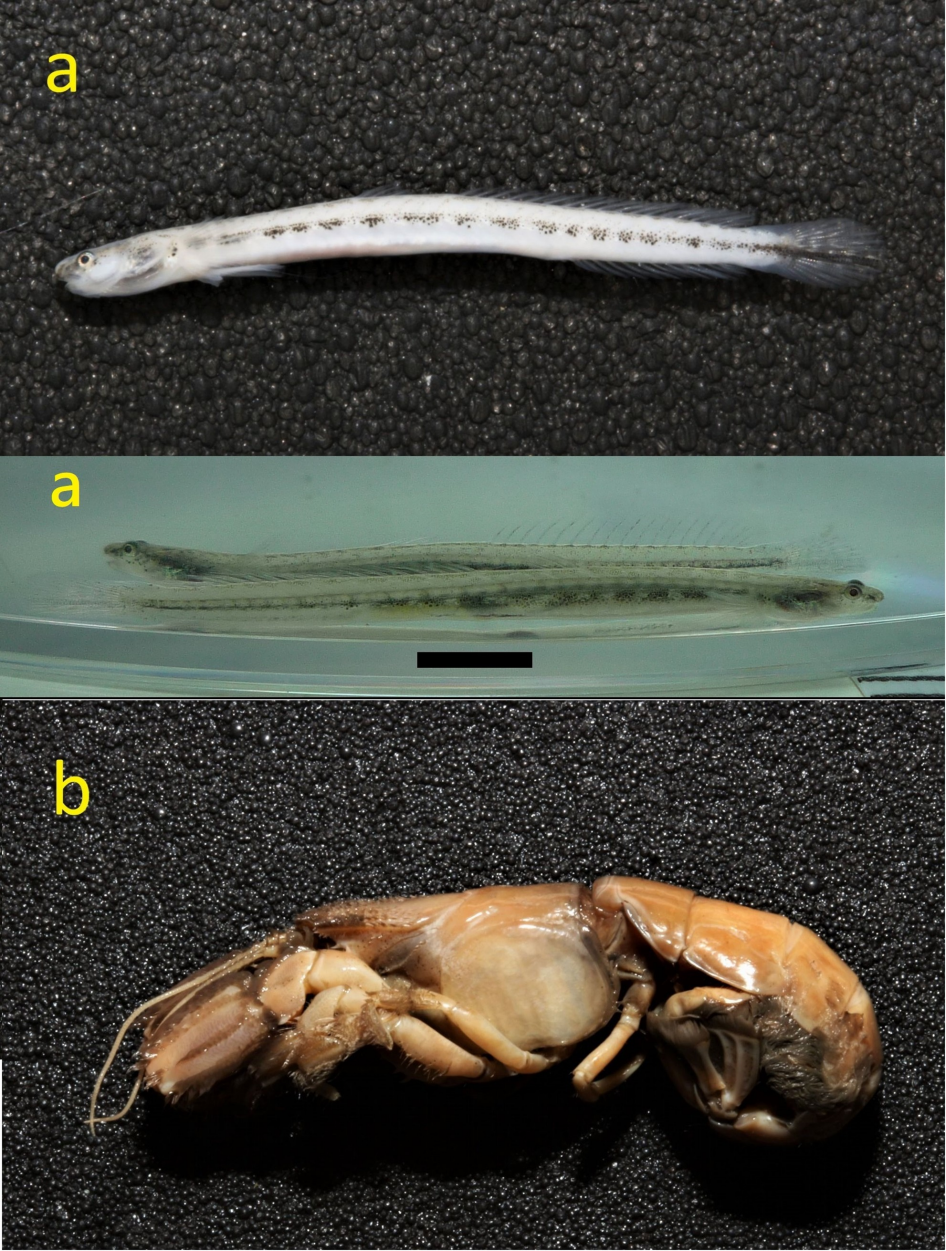
Specimen photograph of *Eutaeniichthys* cf. *gilli* (ASIZP0080765) (a), the scale bar represents 10 mm; and its symbiotic mud shrimp species (*Austinogebia edulis*) (b). Standard length for *E*. cf. *gilli* is 31.2 mm; carapace length for mud shrimp is 18.1 mm.

The mud shrimp, *A*. *edulis* (Fig 1), is an economic species and provides a traditional food in central western Taiwan [8]. It collects the fine sediment particles to build its burrow and perhaps utilizes the organic materials from the burrow wall [9]. Reports found the symbiotic alpheid shrimp *Chelomalpheus crangonus* Anker, Jeng & Chan, 2001 [10], and the squillid shrimp *Cloridopsis scorpio* (Latreille, 1828) [11] from the burrows of *A*. *edulis*. Previous studies showed the bio-accumulation of different heavy metals takes place in the shrimp from nearby wetlands of Changhua Coastal Industrial Park [12]. A recent report found that they are sensitive to cadmium pollution [13]. As yet, the biology and ecology of the mud shrimp *A*. *edulis* remains unstudied [10].

During a program of biodiversity survey and investigation, the biology of the mud shrimp, *A*. *edulis*, the shrimp was found periodically from June 2016 to June 2018 in western Taiwan. Some small slender gobies were found from the tunnel burrowed by *A*. *edulis*. Subsequently, these gobies were identified as *Eutaeniichthys gilli* Jordan & Snyder, 1901 based on their specific features (Fig 1A). This species is a new record of the genus in Taiwan. Previous literatures revealed that the distribution of *E*. *gilli* was mostly in the northern waters of the East China Sea, e.g., Japan, Korea, Yellow Sea and Bohai Sea. Because a thorough phylogenetic analysis is lacking as yet, *Eutaeniichthys* cf. *gilli* was studied here in this respect.

In this study, we report detailed morphological features, including morphometric, meristic, and cephalic sensory papillae of *E*. cf. *gilli* from Taiwan. In addition, a preliminary exploration of its hiding tunnel structure and symbiotic species were analyzed and discussed. Finally, the possible dispersal pathway leading to the present biogeographic distribution of *E*. *cf*. *gilli* in Japan, Korea and extending to western Taiwan are discussed in the present study.

## Materials and methods

### Field sampling and resin casting

**Fig 2:**
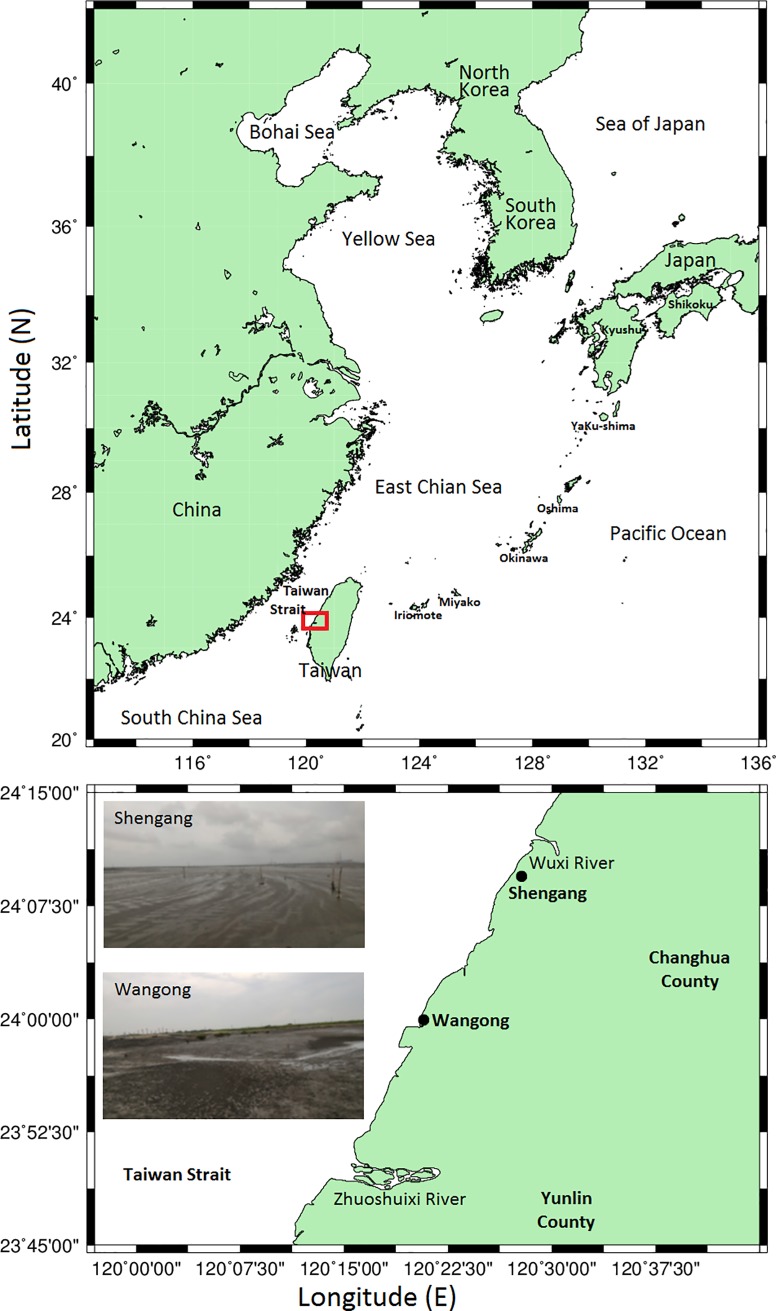
Map of the sampling area in western Taiwan during the investigation period (June 2016 to September 2018).

A total of 56 individuals of *E*. cf. *gilli* were collected from the areas of Shengang (24.168094 ^o^N, 120.457894 ^o^E) and Wangong (23.968126 ^o^N, 120.323173 ^o^E) in western Taiwan (Fig 2). Five lots were collected: 8 individuals on June 1, 2016; 7 individuals on November 3, 2016; 12 individuals on February 22, 2017; 6 individuals on June 13, 2018; and 23 individuals on September 21, 2018. The sites were characterized as mudflat areas dominated by the mud shrimp *A*. *edulis* [12]. Samples of goby *E*. cf. *gilli* were collected carefully from mud shrimp burrows by using a shovel and hand net (mesh size: 0.5 mm) during low tide period. *Eutaeniichthys* cf. *gilli* always appeared at the surface of the pool with turbid underground seawater when digging more than 60 cm deep.

The resin casting method has been applied to study the burrow of mud shrimps [14–16]. Previous researchers found that mud shrimps could be fixed in the resin. Therefore, we used this method to find evidence of the existence of *E*. cf. *gilli* in the burrows of mud shrimps. A total of 30 resin casts were obtained from Shengang and Wangong from 2016 to 2018 using polyester resin. The liquid resins were poured into the mud shrimp burrows, and retrieved after 24 hours when they turned into a solid cast model.

### Sample treatment

Specimens were fixed in 10% formalin for three days, and then transferred to 70% ethanol for long-term preservation. The morphological measurements follow Miller [17] and the meristic analysis followed Chen and Shao [18]. The terminology of cephalic sensory canals and free neuromast organ (sensory papillae) is based on Sanzo [19], and Huang et al. [20]. All lengths were standard lengths (SL). Examined specimens were deposited at the Biodiversity Research Museum, Biodiversity Research Center, Academia Sinica, Taipei, Taiwan (ASIZP).

### Cultivation in the laboratory

In the laboratory, *E*. cf. *gilli* were cultured in fresh seawater with air supply at a photoperiod of 12:12 hour light/dark. Live *Artemia franciscana* Kellog, 1906 nauplii, copepods, *Pseudodiaptomus annandalei* Swell 1919 and *Apocyclops royi* (Lindberg, 1940) were provided twice a day (at 11:00 and 15:00 h). This was according to a suggestion by Dôtu [21] who reported that *E*. *gilli* feeds on small-sized crustaceans and other organic matter. Seawater quality was maintained at salinity ranges of 25.0–30.0 PSU knowing that it is a brackish water species [22, 23]. Seawater temperature was maintained at 24–26°C as this was measured on the mud surface near the mud shrimp burrow on June 1, 2016. Our experience showed that *E*. cf. *gilli* can successfully survive for more than one year in the laboratory at these conditions.

### Image of the sea surface temperature

The images of the Group for High Resolution Sea Surface Temperature Pilot Project (GHRSST-PP)/ Operational SST and Sea Ice Analysis (OSTIA) were obtained from the Fisheries Research Institute, Council of Agriculture, Taiwan. The images of the sea surface temperature were used for discussing the relationship between water mass transport and the biogeographic distribution of *E*. cf. *gilli*.

## Results

### Historical records of Eutaeniichthys gilli

*Eutaeniichthys gilli* (Japanese name: Himohaze) inhabits sandy-muddy mud, muddy sediment, and tide pools under stones in estuaries [24]. It is distributed in the northwest Pacific region, in temperate waters. This species has been reported in several areas of the coast and estuaries in Japan [21–22, 25–40]; in the Yellow Sea-Masan [41]; in the Yellow Sea and Bohai Sea of Korea [42, 43]; and in the Gulf of Chihli (Bohai) [44], in the Yellow Sea and Bohai Sea of China [45–47].

### Morphological traits of the examined specimens

ASIZP0080765, 5 specimens, 26.5–31.2 mm SL; collected from Shengang and Wangong, western Taiwan, June 1, 2016.

*Diagnosis*. Has a low body and a rather slender head which is relatively small. The upper lip is obviously more prominent than the lower lip. The first dorsal fin rays III, second dorsal fin rays I/16–17, anal fin rays I/11, pectoral fin rays 14–15. Second dorsal fin and anal fin base are long. The body is covered with numerous tiny scales. All scales are tiny and scattered, and scales never touch adjacent scales, and are never arranged in a line. The lateral sides of the body with about 16–18 distinct grayish black blotches, dorsal side with 16–17 cross bars, dorsal side of neck with two longitudinal black stripes, forward extending to the interorbital region. Caudal fin has a distinct longitudinal black stripe, starting from the caudal fin base, extending to the rear end.

*Description*. Morphometric measurements are given in Table 1. Body low and rather slender, sub-cylindrical anteriorly and compressed posteriorly. Head is moderately small, eye high and large; upper lip obviously more prominent than lower lip; and mouth oblique, corner of mouth extending to a vertical line with the posterior margin of the pupil. Anterior nostril is a short tube and posterior nostril is a round hole. Gill-opening restricted, extending ventrally to a vertical line of one-third of the posterior operculum margin.

**Table 1:** Morphometric measurements of *Eutaeniichthys* cf. *gilli* from Taiwan.

	Males (n = 3)		Females (n = 2)	
Percent of the standard length (%)				
Head length	16.3–17.9	(17.0)	17.7–18.1	(17.9)
Predorsal length	41.3–42	(41.7)	39.7–40.4	(40.0)
Snout to 2nd dorsal origin	54.5–56.8	(55.8)	53.6–57.1	(55.3)
Snout to anus	64.9–67	(65.9)	67.5–69.1	(68.3)
Snout to anal fin origin	69–69.8	(69.4)	70.6–71.3	(70.9)
Prepelvic length	18.1–19.2	(18.5)	19.1–19.6	(19.4)
Caudal peduncle length	10.1–10.6	(10.4)	9.8–10.3	(10.0)
Caudal peduncle depth	4.5–4.9	(4.7)	4.9–5	(4.9)
1st dorsal fin base	6.4–6.9	(6.7)	6.4–6.8	(6.6)
2nd dorsal fin base	37.8–38.3	(38.0)	37.2–37.4	(37.3)
Anal fin base	19.9–21.2	(20.5)	20.8–20.9	(20.8)
Caudal fin length	15–16	(15.6)	15.5–17	(16.2)
Pectoral fin length	10.9–11.5	(11.3)	11.3–11.7	(11.5)
Pelvic fin length	11.8–12.2	(12.0)	11.6–11.7	(11.7)
Body depth at pelvic fin origin	6.3–6.7	(6.5)	6–6.4	(6.2)
Body depth at anal fin origin	5.9–6.3	(6.1)	6–6.7	(6.4)
Body width at anal fin origin	3.5–3.8	(3.7)	3.5–3.8	(3.7)
Pelvic fin origin to anus	47.8–50	(49.1)	51.4–51.7	(51.6)
Percent head length (%)				
Snout length	16.1–17	(16.6)	14.9–15.7	(15.3)
Eye diameter	16.1–19.1	(18.0)	19.6–21.3	(20.4)
Cheek depth	12.5–14.9	(14.0)	10.6–11.8	(11.2)
Postorbital length	64.3–68.8	(67.0)	64.7–66	(65.3)
Head width, maximum	41.1–42.6	(41.8)	35.3–36.2	(35.7)
Head width at upper gill	32.1–34	(33.2)	31.4–31.9	(31.6)
Bony interorbital width	10.4–12.8	(11.3)	9.8–10.6	(10.2)
Fleshy interorbital width	17.9–19.1	(18.6)	17–17.6	(17.3)
Lower jaw length	28.6–31.9	(29.9)	27.5–27.7	(27.6)

*Coloration in alive specimens* (Fig 1).—Head and body pale yellowish white, body side with about 16–18 distinct grayish black blotches, some blotches are slightly fused. Dorsal side has 16–17 cross bars, starting from neck, and extending to the caudal peduncle. Dorsal side of neck has two longitudinal black stripes, which are extending forward to the interorbital region. Cheek and opercular region has some black spots and bars. The pectoral fin base has some black spots. Dorsal fin, pectoral fin, anal fin, and pelvic fin are without any spot. Caudal fin has a distinct longitudinal black stripe, starting from the caudal fin base, and extending to the rear end.

*Head canals*.—Head pores absent.

**Fig 3:**
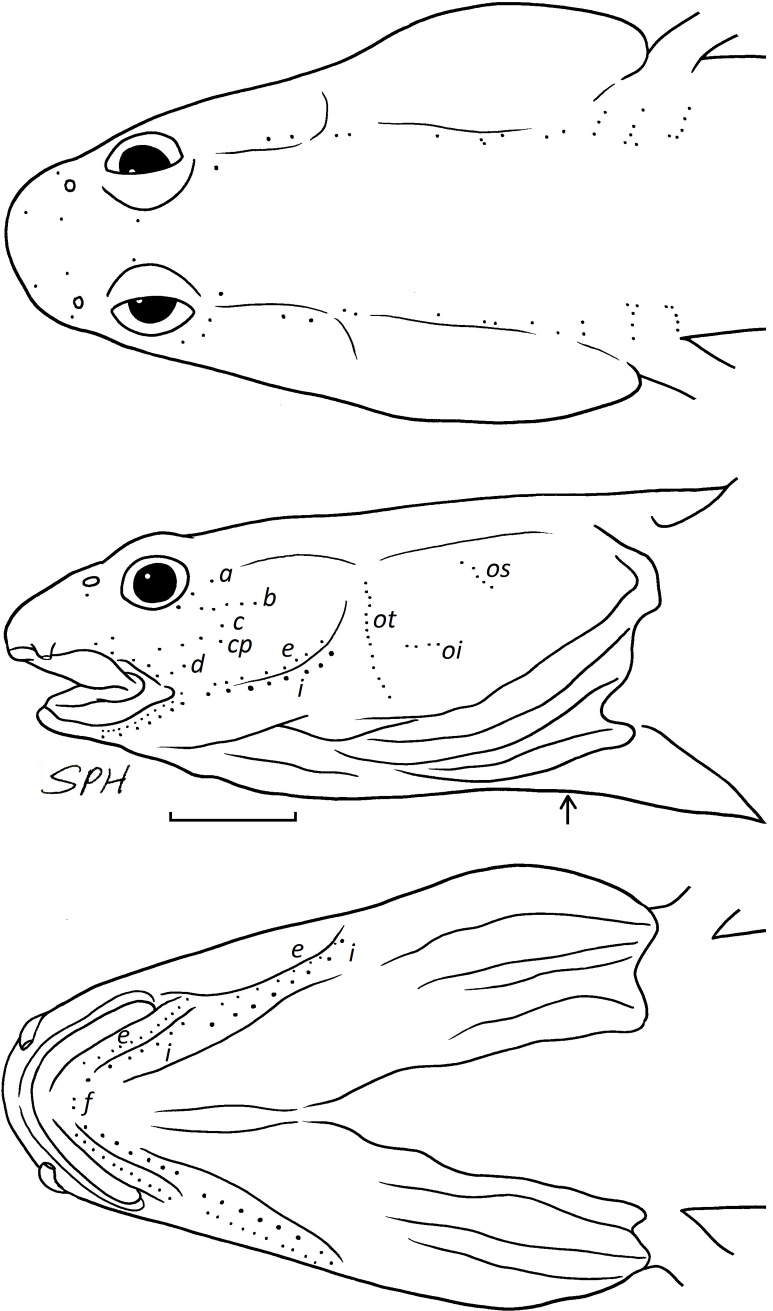
Cephalic sensory papillae of *Eutaeniichthys* cf. *gilli*. ASIZP0080765, 31.2 mm SL. Arrowhead indicate the gill opening, scale bar = 1mm.

*Sensory papillae* (Fig 3).—Row *a* short, about two-thirds of orbit diameter; row *b* long, about three-fourth of orbit diameter, starting from the vertical of rear margin of the orbit; row *c* approximately equivalent to orbit diameter. Single *cp* papilla is located under the last papilla of row *c*. Row *d* particularly short, about half of orbit diameter. Opercular papillae with rows *ot*, *oi*, and *os*, rows *ot* and *oi* well separated. Row *f* consists of a pair of single papillae.

*Fins*.—First dorsal fin rays III, second dorsal fin rays I/16–17 (modally 17), anal fin rays I/11, pectoral fin rays 14–15. The first dorsal fin is small and rounded, fin spine II longest. Second dorsal fin base is long, its rear tip of ray extending to the anterior margin of the procurrent caudal ray when depressed. Anal fin base long, its origin inserts below sixth branched soft ray of second dorsal fin. Pectoral fin is broad and rounded. Pelvic fin disc rounded. Caudal fin fan-shaped, the rear edge rounded.

*Scales*.—Body covered with numerous tiny scales, all scales tiny and scattered, scales never touch adjacent scale, and is never arranged in a line. Predorsal area and belly also covered with tiny scales. Operculum, preoperculum, prepelvic areas, and pectoral fin base are always naked.

### Resin casts of burrows trapping symbiotic species

**Fig 4:**
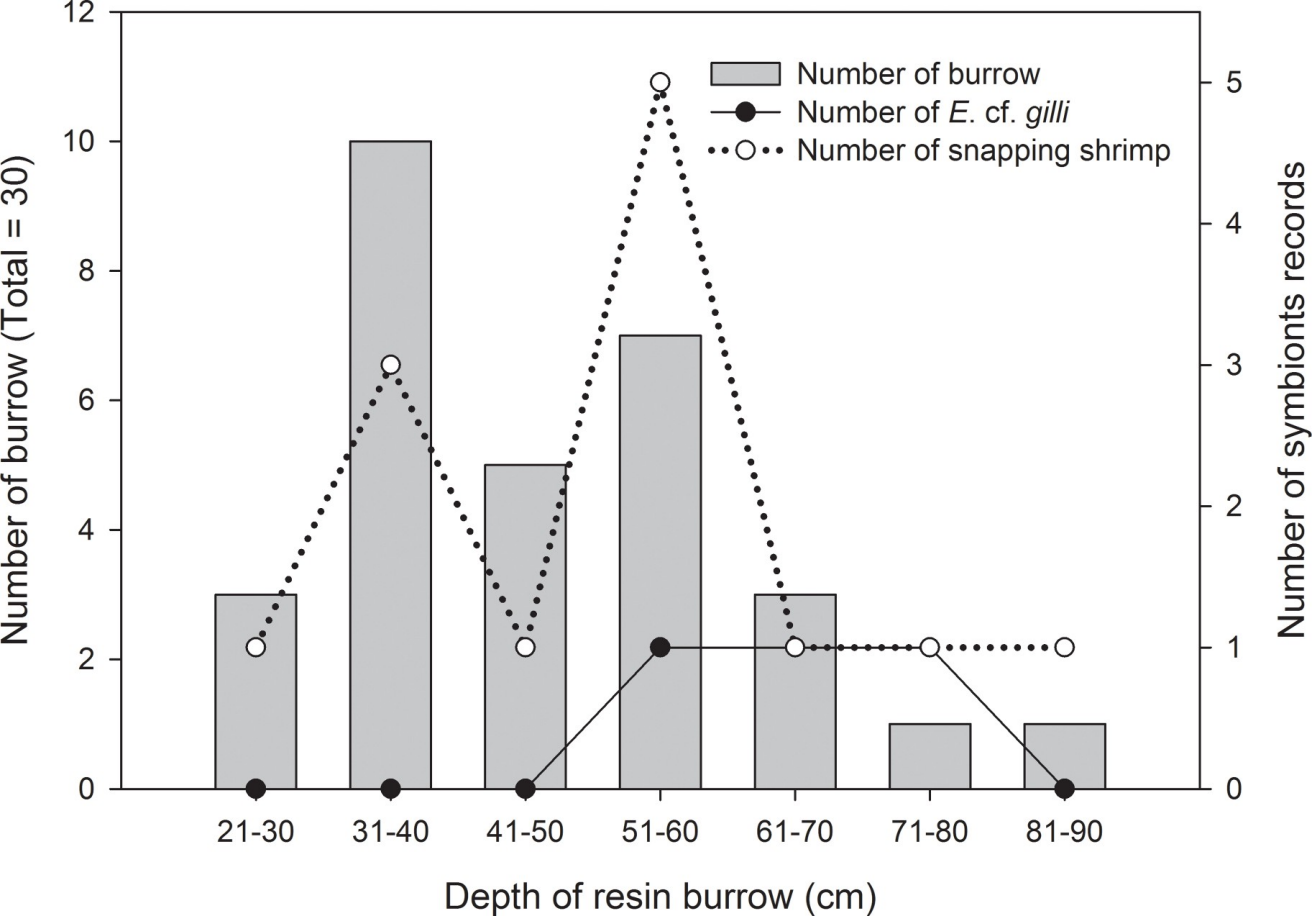
The distribution of the depth of the mud shrimp burrow, and the number of the *Eutaeniichthys* cf. *gilli* and snapping shrimp appeared in the burrow of different depths.

**Fig 5:**
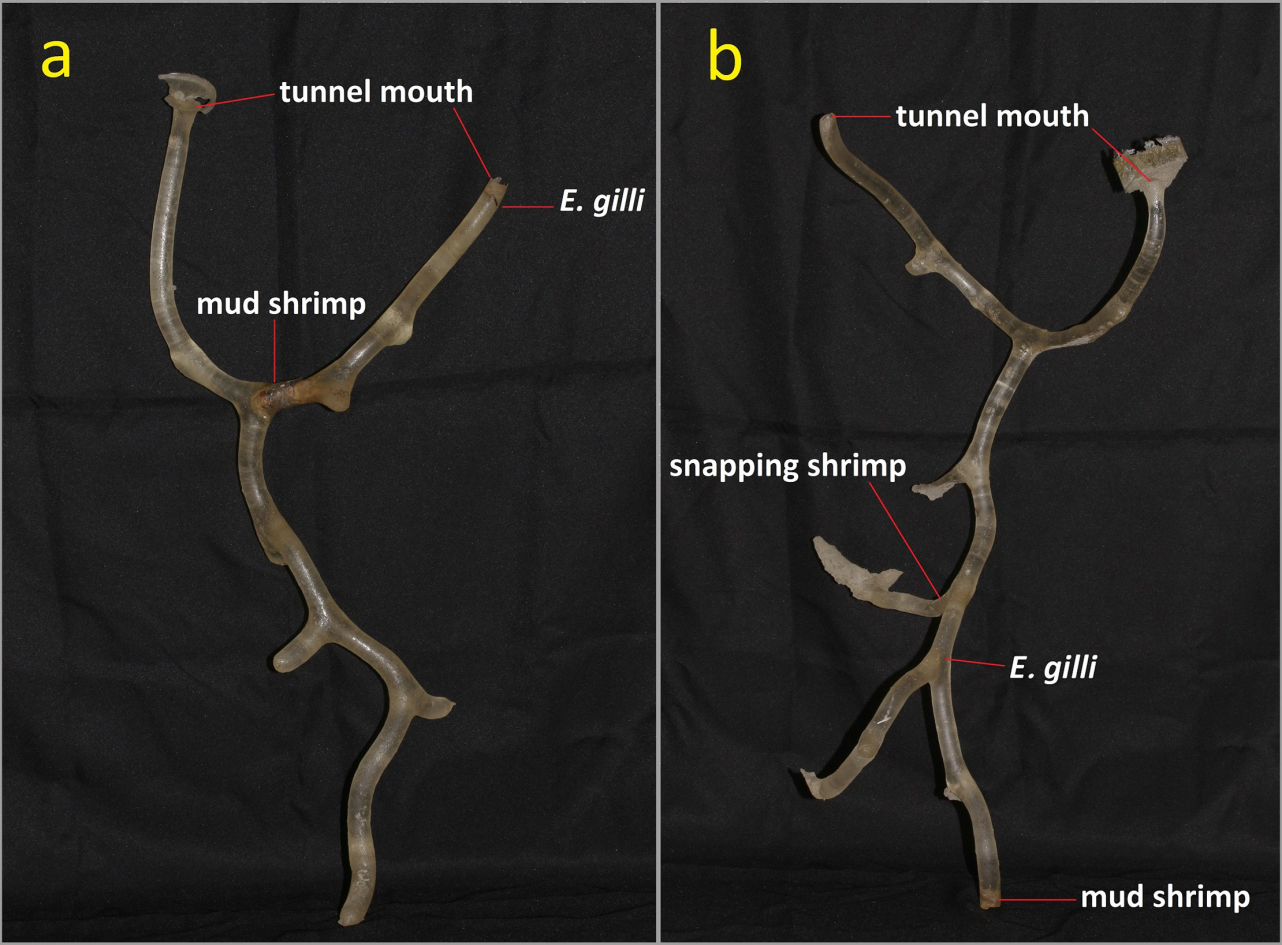
Two examples of tunnels burrowed by mud shrimp (*Austinogebia edulis*). Exact location for the symbiotic species included *Eutaeniichthys* cf. *gilli*, *A*. *edulis* and snapping shrimp are labeled. Straight heights of tunnels are 68.2 cm (a), and 73.0 cm (b).

A total of thirty resin cast burrows of the mud shrimp *A*. *edulis* were fixed and examined in this study. Among all resin cast burrows, *Eutaeniichthys* cf. *gilli* and snapping shrimp (unidentified) were recorded from 3 (10%) and 13 (43.3%) burrows, respectively (Fig 4). *Eutaeniichthys* cf. *gilli* were only recorded in burrows between 50 and 80 cm in length, with only one individual in a burrow, whereas the snapping shrimp recorded in all depth of resin burrows. Two classic examples of burrows were shown in Fig 5. *Eutaeniichthys* cf. *gilli* were recorded at places near the tunnel mouth (Fig 5A) and at midlength of depth (Fig 5B) in mud shrimp burrows.

### Distribution and habitat in Taiwan

So far, the present report is the first record of this species from sandy and muddy bottoms of the intertidal zone in Shengang and Wangong, western Taiwan. There is no observation that *E*. *gilli* can dig the inhabited burrow themselves. Instead, the mud shrimp *A*. *edulis* is known to construct a strong burrow structure that can change the composition of the sediment [9]. We conclude, therefore, that *E*. cf. *gilli* makes use of the burrow as a sheltered habitat that was built by the mud shrimp, *A*. *edulis*.

## Discussion

The present study identified *E*. cf. *gilli* inhabiting a wetland near the industrial park and hiding in mud shrimp burrows. This species belongs to the family Gobiidae, which is a highly diverse bony fish family worldwide [48]. However, gobiids are important but frequently misidentified and their biology and ecological niche are poorly understood [49]. There are about 247 species from 77 genera in the gobiid family in Taiwan [50]. Historical records of the goby, *E*. cf. *gilli*, along the coast of Taiwan are not available. This new record for Taiwan is thus the southernmost record of the species in the world. However, *E*. *gilli* in the area of southern Ryukyu is still inadequately known, and our study is the first to document *E*. cf. *gilli* in waters of this region. Previous *in situ* studies found that *E*. *gilli* utilized mud shrimp burrows as a breeding habitat during the period from May to August [21]. A laboratory study found that *E*. *gilli* spent 25 to 50% of its time in the burrow of the mud shrimp [33]. The burrow of mud shrimp is an important habitat that *E*. *gilli* utilized as a nesting site for spawning and deposition of eggs in the shallow tidal creek of Obitsu-gawa River estuary [31]. The *E*. *gilli* takes shelter inside the burrows of *A*. *edulis* which benefits their reproduction.

All previous studies found that *E*. *gilli* is distributes in coastal areas of Kyushu, Japan, the Yellow Sea, and the Bohai Sea of Korea and China. These records of distribution areas were north of 25 degrees northern latitude [21–22, 25–47]. The distribution of *E*. *gilli* was related with the Tsushima Current in an earlier report [42]. The distance from the Yellow Sea and Kyushu to the present study area is about 1400 to 1500 km. The China Coastal Current (CCC) of the Yellow Sea and the Bohai Sea flows along northern Taiwan into the eastern Taiwan Strait during the period in November to April when the northeast monsoon prevails [51]. The water mass transport of the CCC might be the major contributor to the geographic dispersal and distribution patterns of *E*. cf. *gilli*.

**Fig 6:**
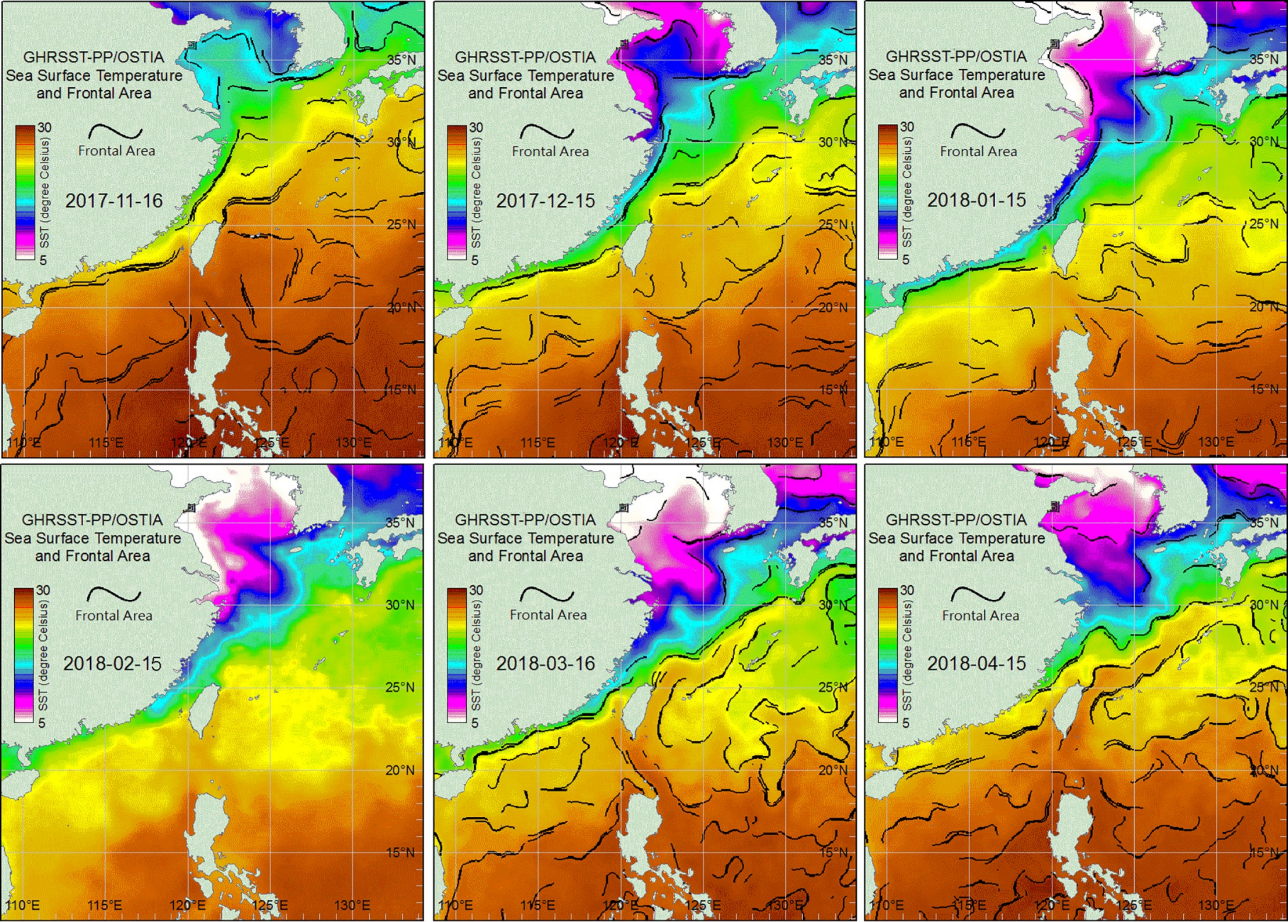
The sea surface water temperature images from mid-November 2017 to mid-April 2018 derived from GHRSST-PP/OSTIA demonstrates the water mass distribution in the East China Sea, the Taiwan Strait, and the South China Sea.

Several studies reported that the water masses of the CCC plays an important role in transporting marine creatures to coastal waters of Taiwan, such as copepods [52], jellyfish [53–55], zooplankton in general [56], and affects the communities of macroalgae [57]. The calanoid copepod, *Calanus sinicus*, provides an example that demonstrates that the CCC drives it from the Yellow Sea and the Bohai Sea to the southern East China Sea area, and to Hong Kong [58]. Sea water temperature images provided by GHRSST-PP/OSTIA from mid-November 2017 to mid-April 2018 demonstrated the cold water mass distribution from the Yellow Sea, the Bohai Sea, along South Korea, Japan and extending southward to the Taiwan Strait where the specimens were collected during the present study (Fig 6). Therefore, the present results suggest that the dispersal and biogeographical distribution of *E*. cf. *gilli* are caused by the water mass transport of the CCC, which continues towards Hong Kong and Vietnam. The biogeographic distribution of the mud shrimp, *A*. *edulis*, extends from north of Taiwan towards south of Vietnam [8]. Therefore, it has to be studied whether the distribution range of *E*. *gilli* extends to even Vietnam in the South China Sea.

Previous investigations have shown the utility of burrows of upogebiid and callianassid shrimps by *E*. *gilli* [21, 22, 59]. The records show that *E*. *gilli* has a symbiotic relationship with the mud shrimp *Upogebia major* [60], and with *Upogebia yokoyai* [33, 34, 59] in Japan. Shrimp burrows are used by several gobies for symbiotic associations [61]. The records found more than 120 gobiid fishes in symbiosis with alpheid shrimps; the shrimps built burrows and share those with gobies as a refuge providing shelter, gobies give feedback on this symbiotic relationship with a warning signal when predators appear [62]. The mutual association is beneficial to one partner, or both shrimp and goby [63]. The present study is the first to report the associations of *E*. cf. *gilli* with the mud shrimp *A*. *edulis* burrows worldwide. The records show that the symbiotic hosts of *E*. *gilli* can be diverse and are not specific. This association would allow a sympatric coevolution and biogeographic codistribution of goby *E*. cf. *gilli* and its host (*A*. *edulis*).

The resin casting method has been applied to study the burrow architecture of different mud shrimps, such as *Austinogebia edulis* [16], *Upogebia major* [15], and *Upogebia omissa* [64]. In general, resin casting of mud shrimp burrows showed that they were approximately Y-shaped, with an upper U-part [15, 16, 64]. The present study applied this method to catch *E*. cf. *gilli* in burrows of the mud shrimp. The results found that the occurrence rate of *E*. cf. *gilli* was 10%. The goby prefers burrows with depth longer than 50cm. Observations in the laboratory found that the behavior of *E*. cf. *gilli* was very agile. They might easily escape from through the other opening of the burrow. Designing a suitable method to avoid underestimating the number of *E*. cf. *gilli* populations and those inhabiting burrows will be a necessary future task.

Only a few studies have measured the lengths of *E*. *gilli*. Lengths records range from newly hatched larvae being 3.6 mm [21] to a maximal length of adults with 50 mm [65] in the waters of Japan. The values of the lengths among adult *E*. *gilli* are similar to those observed in the present study (Table 2). Results of previous reports are insufficient to compare possible geographic differences of lengths in *E*. *gilli*. Body lengths measurements of *E*. cf. *gilli* larvae could not be done in the present study since larvae were lacking from the samples in the present study. The spawning period of *E*. cf. *gilli* in western Taiwan needs to be confirmed by more frequent collections.

**Table 2:** Review of body length in different stages of *Eutaeniichthys gilli* collected from Japan and in the present study.

Body length	Stage	Reference
50 mm	Actual specimen	Tomiyama [65]
3.6 mm	Newly hatched larvae	Dôtu [21]
Maximum body size 4 cm	Actual specimen	Nakabo [68]
8.9–10.6 mm	Immature-fish	Inui et al. [29]
5.5 mm—35.1 mm	Larvae—adult	Hermosilla et al. [30]
18.8–34.8 mm	Actual specimen	Hermosilla et al. [31]
23.6–42.6 mm	Actual specimen	Matsui et al. [35]
32.3 mm	Actual specimen	Kanagawa et al. [40]
25–38 (33.9 ± 3.73) mm	Actual specimen	Present study

Remarkably, all *E*. cf. *gilli* examined in this study were collected from mudflat near the Changhua Coastal Industrial Park. This indicates that the species can tolerate a rather polluted environment. The population size of *E*. *gilli* is critical here since it affects the endangered status of the species as it is indicated in the Red List in Japan [66], as threatened and near-threatened goby species [34, 35, 37], rare species [38] and vulnerable species in the tidal estuarine systems [40]. Currently, there are 102 fish species reported in the endangered list in Taiwan [50]. The present report is the first record of this species in mudflats in western Taiwan. Biological and ecological investigations need more follow-up support. Proven habitat need to consider the interspecific differences in environmental factor preference and tolerance of gobies inhabiting tidal flats [67]. The protection of suitable habitats will be an important biodiversity conservation measure. Since mudflats are often threatened by landfilling, drilling for fossil energy sources in rivers, and sedimentary habitat changes due to changing tidal and riverine currents, they are particularly vulnerable and need a sustainable conservation management measure.

## Conclusion

This investigation documents a unique and rare symbiotic goby *E*. cf. *gilli* associated with the mud shrimp *A*. *edulis* from the mudflat in western Taiwan. The population size and biogeographic distribution of *E*. cf. *gilli* in Taiwan needs to be studied in details in the future to provide its sustainable conservation. Furthermore, previous records demonstrate that *E*. cf. *gilli* is distributed in the marine waters of China, Korea, Japan, and the present it report from mudflats of the western Taiwan coast. Studies of the phylogenetic relationships of *E*. *gilli* are needed to better understand their population differentiation and geographic distribution.

## Supporting information

S1 File Supporting information file provides information of distribution of the depth of the mud shrimp burrow, and the number of the *Eutaeniichthys* cf. *gilli* and snapping shrimp appeared in the burrow of different depths (Fig 4). (XLSX)
